# Enhancing effect of β-elemene emulsion on chemotherapy with harringtonine, aclacinomycin, and Ara-c in treatment of refractory/relapsed acute myeloid leukemia

**DOI:** 10.12669/pjms.306.5207

**Published:** 2014

**Authors:** Cuiping Zheng, Xiaoping Cai, Shenghao Wu, Zhen Liu, Yuejian Shi, Wenjin Zhou

**Affiliations:** 1 Cuiping Zheng, Department of Blood Chemotherapy, The Central Hospital, Wenzhou, 325000, P. R. China.; 2 Xiaoping Cai, Department of Blood Chemotherapy, The Central Hospital, Wenzhou, 325000, P. R. China.; 3 Shenghao Wu, Department of Blood Chemotherapy, The Central Hospital, Wenzhou, 325000, P. R. China.; 4 Zhen Liu, Department of Blood Chemotherapy, The Central Hospital, Wenzhou, 325000, P. R. China.; 5 Yuejian Shi, Department of Blood Chemotherapy, The Central Hospital, Wenzhou, 325000, P. R. China.; 6 Wenjin Zhou, Department of Blood Chemotherapy, The Central Hospital, Wenzhou, 325000, P. R. China.

**Keywords:** β-elemene, chemotherapy, leukemia, refractory/relapsed AML

## Abstract

***Objective*** *:* This study is to determine the curative effect of β-elemene emulsion on chemotherapy in the treatment of refractory/relapsed acute myeloid leukemia (AML).

***Methods*** *:* In the β-elemene emulsion plus HAA chemotherapy (harringtonine, aclacinomycin, Ara-c group) group, 120 cases received β-elemene emulsion (400 mg) plus the HAA treatment. A 14-day treatment was a course of treatment, followed by an 8-14 day pause, and then the next course of treatment. The HAA treatment included Ara-C, 100 mg/m2, once every 12 h from day 1 to day 7; aclacinomycin, 20 mg/m2, from day 1 to day 7; and homoharringtonine, 4 mg/m2, from day 1 to day 7. The patients in the control HAA group received HAA treatment only. For both groups, effective antibiotics were given to patients when it was necessary.

***Results*** *:* The total effective rate in the β-elemene emulsion plus HAA group was 80.8%. But the total effective rate in the HAA only group was 52.9%. These results suggest that the β-elemene emulsion plus HAA treatment has a much better curative effect in comparison with the HAA only treatment (P < 0.05). Furthermore, β-elemene emulsion has slightly adverse response, without causing blood and bone marrow depression.

***Conclusion*** *:* β-elemene emulsion has a curative effect in treatment of refractory/relapsed AML in combination with harringtonine, aclacinomycin, and Ara-c.

## INTRODUCTION

The refractory/relapsed acute myeloid leukemia (AML) has the characters of low remission rate and high fatality rates.^1^^-^^3^ Traditional chemotherapy of refractory/relapsed AML has reliable efficacy, but it often results in serious adverse responses.^4^^,^^5^ β-elemene is an anti-tumor ingredient extracted from a Chinese traditional medicine herb, with inhibitory effects on the synthesis of DNA, RNA, or protein in malignant cells.^6^^-^^9^ β-elemene may decreases expression of NF-κB, COX-2 and COX-2’ product PGE2.^9^^,^^10^ β-elemene improves the apoptosis induction effect of aclacinomycin on leukemic cells.^11^^-^^13^ We have recently found that β-elemene enhances the aclarubicin-induced apoptosis in human leukemia cells in vitro.^14^ In this study, the curative effects of β-elemene emulsion in combination with chemotherapy for treatment of refractory/relapsed AML are reported.

## METHODS

***Patient’s data: ***In the β-elemene emulsion plus chemotherapy group, 120 refractory/relapsed AML cases were enrolled. Among them, there were 35 refractory AML patients, 32 relapsed AML patients, 48 myelodysplastic syndrome-transformed AML patients, and 5 cases of therapy-related AML. There were 84 male cases and 36 female cases. The age range was 25-79.

In the chemotherapy only group, 121 cases were enrolled. The age range was 14-75. Among them, there were 39 refractory AML cases, 31 relapsed AML patients 31, 45 myelodysplastic syndrome-transformed AML patients, and 6 cases of therapy-related leukemia. Diagnostic criteria of the two groups described above were consistent with the reported procedure. Prior written and informed consent were obtained from every patient and the study was approved by the ethics review board of the central hospital, Wenzhou, China.

***Treatments: ***In the β-elemene emulsion plus HAA chemotherapy (harringtonine, aclacinomycin, Ara-c group) group, 120 cases received β-elemene emulsion (400 mg/d) plus the HAA treatment. A 14-day treatment was a course of treatment, followed by an 8-14 day pause, and then the next course of treatment. The HAA treatment included Ara-C, 100 mg/m2, once every 12 h from day 1 to day 7; aclacinomycin, 20 mg/m2, from day 1 to day 7; and homoharringtonine, 4 mg/m2, from day 1 to day 7. The patients in the control HAA group received HAA treatment only. For both groups, effective antibiotics were given to patients when it was necessary.

Before and after therapy, the symptoms were observed and the routine blood and bone marrow examinations were performed. Electrocardiogram and chest CT examinations were performed and functionality of the important organs and conditions of infection were monitored for all of the patients.

***Evaluation of curative effects:*** The curative effects after two courses of treatments were compared between the two groups, the β-elemene + HAA group and the HAA group. The routine blood examination and bone marrow examination were performed. The curative effects, including complete remission (CR), partial remission (PR), non-remission (NR).^15^

***Statistical analysis: ***Statistical analysis was done by SPSS18.0 software. The comparison of two groups was performed with χ2 test. The comparison of adverse response between the two groups was conducted by t test.

## RESULTS

***Curative effects of β-elemene: ***Among the 120 AML cases of the β-elemene emulsion + HAA chemotherapy group, there were 85 cases of CR, 12 cases of PR, and 23 cases of NR. The CR rate was 70.8% (85/120) and the total effective rate was 80.8% (97/16). Among 121 cases of the HAA group, there were 53 cases of CR, 11 cases of PR, and 57 cases of NR. The CR rate was 43.8% (53/121) and the total effective rate was 52.9% (64/121). The curative effects of β-elemene among the two groups were analyzed by the χ2 test, indicating that there were significant differences (P < 0.05) among these two groups (Table-I).

**Table-I:** Efficacy comparison between the β-elemene emulsion plus HAA chemotherapy group and the HAA chemotherapy group

**Groups**	**Cases**	**CR(%)**	**PR(%)**	**NR(%)**	**Effectiveness rate (%)**
β-elemene + HAA	120	85(70.8)	12(10.0)	23(19.2)	80.8
HAA	121	53(43.8)	11(9.1)	57(47.1)	52.9

***Adverse responses: ***The rate of the IV bone marrow depression was 95.8% in the β-elemene emulsion + HAA group (115 cases in 120 cases), Incidence of infection was 93.3% (112 cases in 120 cases). In the HAA only group, the rate of IV bone marrow depression was 97.5% (118 cases in 121 cases) and the incidence of infection was 98.3% (119 cases in 121 cases). The differences in bone marrow depression and infection rates between the two groups were not significant (P < 0.05). No severe kidney function damages were found from patients in both groups. The influence of β-elemene emulsion on blood and bone marrow was given in Table-II, which suggest that β-elemene emulsion does not result into obviously adverse effects on blood and bone marrows.

**Table-II:** Effects on blood and bone marrow of the β-elemene emulsion plus HAA treatment

	**Blood**			**Bone marrow**	
	**Hb g/L**	**WBC (×109/L)**	**PLT (×109/L)**	**Proliferation degree**	**Original cells**
Before therapy	75 ± 15	13.5 ± 0.5	35 ± 10	Low-very active	75 ± 10
After therapy	85 ± 15	4.5 ± 1.0	80 ± 25	Active-very active	3 ± 50

**Note:** Hb, Hemoglobin; WBC, white blood cell; PLT, Blood platelet.

## DISCUSSION

In this study, we treated 120 refractory/relapsed AML cases with β-elemene emulsion plus HAA and 121 refractory/relapsed AML cases with HAA only. The total effective rate in the β-elemene emulsion plus HAA group was 80.8%. But the total effective rate in the HAA only group was 52.9%. These results suggest that the β-elemene emulsion plus HAA treatment has a much better curative effect in comparison with the HAA only treatment. We found that β-elemene emulsion plus HAA treatment had little inhibitory effects on bone marrow^8^^-^^10^^,^^16^ and adverse response.^10^ No patient with bone marrow depression was found among the 120 cases of the β-elemene emulsion plus HAA group. Therefore, it is possible that β-elemene emulsion might be used combined with chemotherapy as the drug of refractory/relapsed AML, especially in those cases of low bone marrow hyperplasia, low hemogram and not suitable for chemotherapy.

β-elemene emulsion has relatively fewer adverse responses. The major side effect is phlebitis, because zingiberene, the main ingredient of β-elemene emulsion, has major stimulus to blood vessel. On the other hand, after emulsifier handling, drug adheres to the blood vessel. The incidence of phlebitis was obviously reduced after 25% magnesium sulfate injection. That is because magnesium ion can directly expand the muscle of peripheral vessels to reduce harmful stimulation. In this study, several patient had slight high fever to be detected, but the fever did not influence the next-round therapy after simple treatment. Because of the limited case numbers in this study, it is not sure whether β-elemene emulsion influences patients’ long-term survival rate. 
